# Signatures of hot carriers and hot phonons in the re-entrant metallic and semiconducting states of Moiré-gapped graphene

**DOI:** 10.1038/s41467-023-37292-4

**Published:** 2023-03-17

**Authors:** Jubin Nathawat, Ishiaka Mansaray, Kohei Sakanashi, Naoto Wada, Michael D. Randle, Shenchu Yin, Keke He, Nargess Arabchigavkani, Ripudaman Dixit, Bilal Barut, Miao Zhao, Harihara Ramamoorthy, Ratchanok Somphonsane, Gil-Ho Kim, Kenji Watanabe, Takashi Taniguchi, Nobuyuki Aoki, Jong E. Han, Jonathan P. Bird

**Affiliations:** 1 Department of Electrical Engineering, University at Buffalo, the State University of New York, Buffalo, NY 14260 USA; 2 Department of Physics, University at Buffalo, the State University of New York, Buffalo, NY 14260 USA; 3 Department of Materials Science, Chiba University, Inage-ku, Chiba, 263-8522 Japan; 4 High-Frequency High-Voltage Device and Integrated Circuits Center, Institute of Microelectronics of Chinese Academy of Sciences, 3 Beitucheng West Road, Chaoyang District, Beijing, 100029 PR China; 5 Department of Electronics Engineering, Faculty of Engineering, King Mongkut’s Institute of Technology Ladkrabang, Bangkok, 10520 Thailand; 6 Department of Physics, Faculty of Science, King Mongkut’s Institute of Technology Ladkrabang, Bangkok, 10520 Thailand; 7 School of Electronic and Electrical Engineering and Sungkyunkwan Advanced Institute of Nanotechnology (SAINT), Sungkyunkwan University, Suwon, 16419 Korea; 8 Advanced Materials Laboratory, National Institute for Materials Science, Tsukuba, 305-0044 Japan

**Keywords:** Electronic properties and materials, Electronic properties and devices, Electronic devices

## Abstract

Stacking of graphene with hexagonal boron nitride (h-BN) can dramatically modify its bands from their usual linear form, opening a series of narrow minigaps that are separated by wider minibands. While the resulting spectrum offers strong potential for use in functional (opto)electronic devices, a proper understanding of the dynamics of hot carriers in these bands is a prerequisite for such applications. In this work, we therefore apply a strategy of rapid electrical pulsing to drive carriers in graphene/h-BN heterostructures deep into the dissipative limit of strong electron-phonon coupling. By using electrical gating to move the chemical potential through the “Moiré bands”, we demonstrate a cyclical evolution between metallic and semiconducting states. This behavior is captured in a self-consistent model of non-equilibrium transport that considers the competition of electrically driven inter-band tunneling and hot-carrier scattering by strongly non-equilibrium phonons. Overall, our results demonstrate how a treatment of the dynamics of both hot carriers and hot phonons is essential to understanding the properties of functional graphene superlattices.

## Abstract (web summary)

Significant attention has been devoted to understanding the low-electric-field properties of carriers in moiré graphene, but high-electric-field transport has not been as well explored. Here, the authors find non-monotonic transport behavior at moiré minigaps due to competition between inter-band tunneling and coupling to out-of-equilibrium phonons.

## Introduction

The dynamics of hot carriers in the energy bands of semiconductors have long been exploited in electronic and optoelectronic technology. Notable examples include light-emitting diodes and lasers^1^, and powerful microwave and terahertz sources^2^. By combining different semiconductors in periodic superlattices, artificial band structures can moreover be implemented. This technology has been widely used to realize active optoelectronic devices, with Bloch oscillators^3,4^ and quantum-cascade lasers^5^ providing just a couple of important examples. The recent emergence of atomically thin two-dimensional (2D) materials, combined with the capacity to stack^6^ these materials in multilayered “van-der Waals heterostructures”, has now opened up the possibility of extending traditional concepts of band-engineering to a whole new class of materials and structures. Atomically thin materials offer many natural advantages over their bulk counterparts, including phononic, excitonic, and plasmonic energies that are enhanced by quantum carrier confinement. By combining the best qualities from different 2D materials, the potential exists to enhance the performance of semiconductor technology well beyond its present capabilities.

**Fig. 1:**
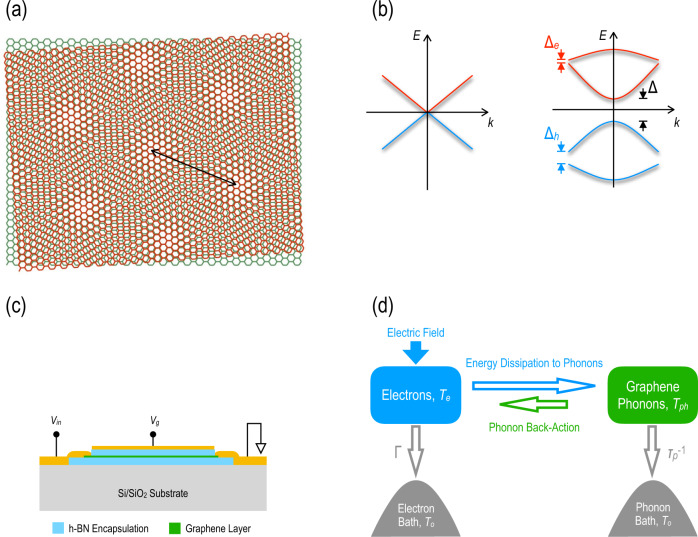
Moiré superlattice of graphene/h-BN. **a** Schematic showing the formation of a Moiré pattern in a heterostructure of two honeycomb crystals with similar lattice constants and a small rotational misalignment. The black arrow denotes the period of the resulting Moiré superlattice. **b** Schematics of the energy bands of monolayer graphene (left) and a graphene/h-BN heterostructure (right). The linear bands observed near the *K*-point in the monolayer are dramatically modified for the heterostructure, with inequivalent gaps appearing at the original Dirac point (Δ) and in the conduction (Δ_e_) and valence (Δ_h_) bands. **c** Schematic illustration of the transistor geometry utilized in our studies. Monolayer graphene is fully encapsulated between thick layers of h-BN and contacted with lithographically designed edge contacts. A top-gate is biased at a variable voltage (*V*_g_) to sweep the Fermi level through the bands of (**b**). The pulsed voltage (*V*_in_) is applied to the input signal line of a coplanar waveguide and the transistor current is measured by feeding it into the 50-Ω input of a fast oscilloscope. **d** The essential elements of our self-consistent theoretical model for electron–phonon energy exchange in graphene. Under non-equilibrium conditions, the electrons and phonons in graphene attain respective temperatures *T*_e_ and *T*_ph_, determined by the strength of the electron–phonon coupling and the loss rates Γ and *τ*_p_^−1^ to electron and phonon reservoirs, respectively. These reservoirs can be taken to be held at some common (fixed) equilibrium temperature (*T*_o_).

Recently, there has been^7^ remarkable progress in realizing new physics in multilayered 2D materials. Graphene and hexagonal boron nitride (h-BN) constitute the workhorse for much of this research, in large part because their honeycomb crystal structures are only slightly mismatched from one another (the lattice constant of h-BN is larger than that of graphene by <2%). Their multilayers can therefore exhibit periodic Moiré structures, whose details depend upon the rotational (or twist) angle between the graphene and h-BN sheets^7–15^ (as indicated in Fig. 1a). The Moiré patterns modify the normally linear dispersion of the graphene bands, opening gaps (see Fig. 1b for a schematic representation) at both the Dirac point (Δ in Fig. 1b) and within the conduction and valence bands^8,10,16^ (Δ_e_ and Δ_h_, respectively). These details have been confirmed by scanning-tunneling microscopy^9^, and from signatures of Hofstadter’s butterfly in quantum-Hall states^17–22^. Elsewhere, the implications of Moiré-miniband formation have also been confirmed in transport experiments performed in magnetic-focusing^23^ and cyclotron-resonance^24^ geometries. Most dramatic of all has been the discovery that, for a “magic” rotational angle of ~1.1°, superconductivity can even be induced in the 2D carbon layer^25^. Collectively, these works reveal an unprecedented capacity to engineer electronic states in 2D heterostructures.

While the band engineering of 2D materials offers enormous promise for the design of tailored electronic systems, realizing this potential requires a proper understanding of hot-carrier dynamics in the artificial bands of their heterostructures. To be clear, this problem of high-field (>kV/cm) transport typically bears little relation to its low-field counterpart. Instead, it is necessary to consider a strong coupling of the electronic and phononic systems, which arises as both are driven far from equilibrium^26^. Yet, while significant attention has been devoted to understanding the low-field (near-equilibrium) properties of carriers in graphene’s Moiré minibands, the corresponding problem of high-field transport has not been as well explored. Only a few studies^26–30^ have addressed how far-from-equilibrium carrier distributions may be generated via optical excitation in graphene/h-BN heterostructures. The number of reports concerning hot-carrier action under strong electrical driving is even smaller^31,32^, and these works do not consider how this transport is modified in the presence of graphene’s Moiré minibands.

In this work, we address the questions identified above by investigating the dynamics of hot carriers in transistors implemented (see Fig. 1c) from graphene/h-BN heterostructures. Using a strategy of fast (~ns) electrical pulsing, we drive the carriers to higher (average) fields (~60 kV/cm) than has previously been possible in this material system. To clarify the influence of the Moiré minibands on the resulting dynamics, we compare the behavior exhibited by two different heterostructures; in one of these, the minibands are well resolved while in the other the usual graphene bands appear to be largely unperturbed. In both of these systems, we find that a proper description of the electrical behavior is only possible if the non-equilibrium nature of both the carrier and phonon distributions, as well as the interactions among them^33^, are taken into account (the essential components of the model are identified in Fig. 1d and its details are described further below). In the system in which the Moiré bands are well developed, we find evidence of reentrant metallic and semiconducting states, which evolve cyclically as the Fermi level is swept through the miniband structure. A metallic increase of resistance with an increasing electric field is found whenever the Fermi level lies within one of the minibands and is explained in terms of scattering from hot phonons at high fields. A more-complicated, non-monotonic, variation of the resistance occurs when the Fermi level is swept through the minigaps, however; we explain this in terms of a competition between electrically driven inter-band tunneling, which determines the response at low electric fields, and coupling to hot phonons that dominates at higher fields. Overall, our results provide important insight into hot-carrier dynamics in graphene/h-BN heterostructures and the impact of hot phonons on their electrical performance.

## Results

### Hot carrier dynamics in graphene without Moiré minibands

**Fig. 2:**
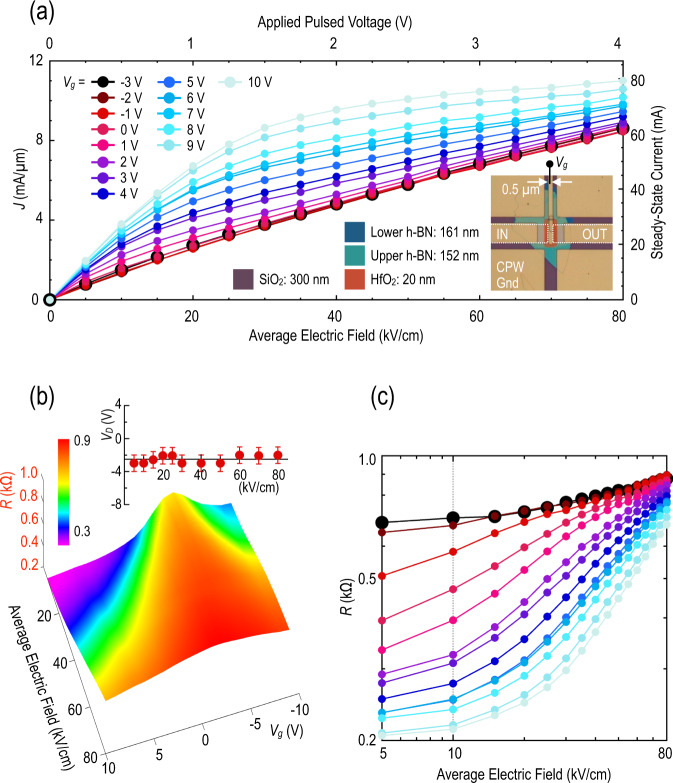
High-field transport in regular graphene bands. **a** Pulsed current-voltage characteristic (plotted as *J* vs. average source-drain electric field) of GhBN1, measured at 300 K. The various curves correspond to different values of *V*_g_, which is incremented in 1-V steps from the Dirac point (*V*_g_ ~ –3 V) to +10 V. Data obtained at the Dirac point are denoted by the larger black circles. The lower-right inset is a false-color optical image of GhBN1, showing the integration of the h-BN/graphene/h-BN device into a coplanar waveguide that is 50-Ω matched for the transient measurements. The white dotted lines in this figure indicate how the contact metallization to the graphene extends to the edges of the top gate (see the “Methods” section for details). **b** Resistance contour constructed from the data of (**a**) (see supplement for details). The resistance is defined as the steady-state transient voltage applied to the heterostructure, divided by the corresponding current (see supplement for details). The inset represents the variation of the Dirac-point gate voltage (*V*_D_) as a function of the (transient) source–drain electric field. The error bars denote the experimental accuracy with which *V*_D_ can be identified from the data. **c** Variation of resistance as a function of the electric field, extracted from the contour of (**b**). Colors shown correspond to those of (**a**). Note the double-log axes of the figure.

We begin by considering the details of hot-carrier dynamics in a fully encapsulated (h-BN/monolayer graphene/h-BN) heterostructure with no clear commensurability between its different layers. In this situation, there is no obvious Moiré-miniband structure and the only role of the encapsulating h-BN is to provide smooth and stable surfaces in contact with the graphene^34^. In this sense, the h-BN forms an idealized dielectric environment for the graphene, free of the unintended charges that plague substrates like SiO_2_. In Fig. 2a, we show (at room temperature) the variation of current density (*J*, obtained by dividing the transient current by the channel width of the transistor of 7.2 μm) with the average source–drain electric field (note that 10 kV/cm = 1 V/μm), as determined by transient pulsing for a heterostructure of this kind (GhBN1, measured with 60-ns transient pulses, see the “Methods” section for details). (The reader should note that the electric fields quoted in this paper are average values, determined by dividing the voltage drop between the source and drain by the distance between—0.5 μm—these contacts. While these averages, therefore, do not account for any nonuniformity of electric field within the channel, we nonetheless consider them useful for comparing the behavior of different devices, as well as the results of different studies in the literature.) By sweeping the voltage (*V*_g_) applied to the top gate (shown in the inset to Fig. 2a) of the device, we can move the Fermi level continuously through the energy bands (in this case the conduction band). The maximum resistance state, indicated by the larger black symbols in the figure, corresponds to the situation where the Fermi level aligns with the Dirac point. Close to this condition, *J* varies almost linearly with the electric field, a behavior that has previously been explained in terms of Landau–Zener tunneling^31^. In this mechanism, an increase of the electric field promotes the tunneling of electrons from filled states in the valence band to empty ones in the conduction band, thereby increasing the number of free carriers involved in transport^35^.

When the gate voltage is used to drive the Fermi level away from the Dirac point the current–voltage curves of Fig. 2a deviate significantly from quasi-linear form, exhibiting instead a tendency towards saturation (for average fields >40 kV/cm). In contrast to previous observations for graphene-on-SiO_2_^36^, yet consistent with prior work on graphene/h-BN^32^, the saturation is never fully complete. Consequently, the current density reaches ultimate values larger than 10 mA/μm (near the highest average field of 80 kV/cm), significantly exceeding the levels reported in previous work. This observation is consistent with the capacity of fast pulsed measurements to attain high current densities, well beyond those realizable in DC measurements, by suppressing self-heating^36–38^ of the system under study.

The data of Fig. 2a may be used to construct a color contour (Fig. 2b) that denotes the variation of the resistance (*R*, determined by dividing the steady stage voltage drop across the device by the corresponding value of the transient current, see Supplementary Information) of the graphene monolayer as a function of electric-field and *V*_g_. At the lowest average field of 5 kV/cm, the Dirac point (defined to occur when *V*_g_ ≡ *V*_D_) can be clearly identified (near *V*_g_ = −3 V) and the relative overall resistance variation induced by the gate voltage corresponds to ~150%. With an increase in the electric field, the size of this variation shrinks and the Dirac peak becomes strongly broadened around its center point. (Importantly, as we indicate in the inset to Fig. 2b, the position of the Dirac point does not change in any systematic way as the pulsed-field strength is increased).

An alternative way to represent the contour of Fig. 2b is to extract curves that plot the variation of *R* as a function of the average electric field. Such a plot is presented in Fig. 2c, for several gate voltages. The resulting curves each exhibit qualitatively similar behavior, with the resistance increasing monotonically with the electric field and merging towards (but not fully reaching) a common variation at the highest fields. Overall, these variations are strongly indicative of the role of hot-electron effects at high electric fields. In fact, as we shall show further below, to properly capture the behaviors revealed in Fig. 2c it will be necessary to invoke the presence of both hot carriers and hot phonons and to account for the interaction that arises among them at high fields.

### Hot carrier dynamics in graphene in the presence of well-formed Moiré minibands

**Fig. 3:**
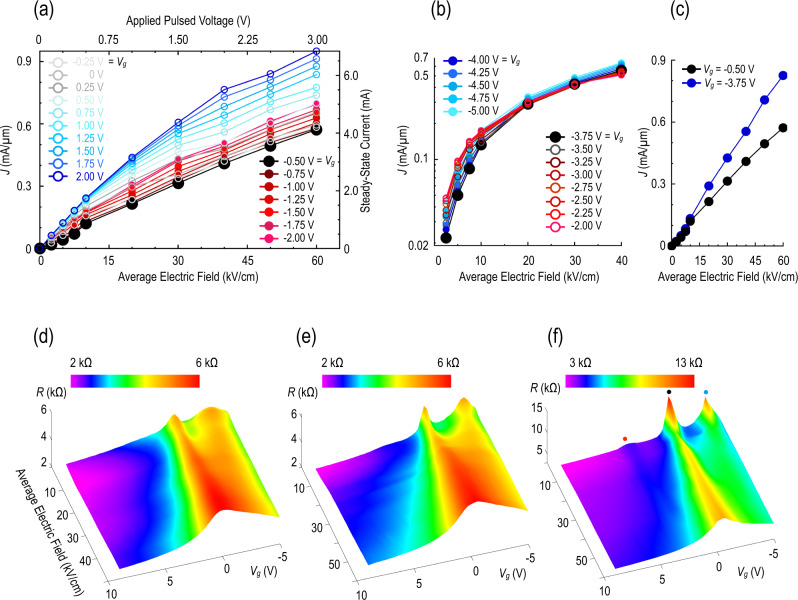
High-field transport in Moiré-gapped graphene. **a** Pulsed current-voltage characteristic of GhBN2, measured at 3 K. The various curves correspond to different values of *V*_*g*_, which is incremented in 0.25-V steps over the range –2.00 V ≤ *V*_g_ ≤ 2.00 V. Data obtained at the Dirac point (*V*_g_ = –0.50 V) are denoted by the larger black circles. **b** Corresponding pulsed current–voltage characteristic of GhBN2, measured at 3 K while varying gate voltage in the range –5.00 V ≤ *V*_g_ ≤ –2.00 V. Note that the current density is plotted on a logarithmic scale for clarity. **c** Comparison of the pulsed current-voltage characteristics of GhBN2, measured at *V*_g_ = –0.50 V and *V*_g_ = –3.75 V. **d** Resistance contour of GhBN2, measured at 300 K. **e** Resistance contour of GhBN2, measured at 77 K. **f** Resistance contour of GhBN2, measured at 3 K. The contours of panels **d**–**f** were constructed from measurements of pulsed current-voltage characteristics, such as those in (**a**) and (**b**).

In this section, we turn our attention to the issue of how the dynamics of hot carriers in graphene are impacted by Moiré miniband formation. In Fig. 3a, we plot the variation of current density with average source–drain electric field for a heterostructure (GhBN2) that manifests strong signatures of Moiré bands. The data shown in this figure correspond to the gate voltage range –2.00 V ≤ *V*_g_ ≤ 2.00 V, for which the minimum current level is seen to occur near *V*_g_ = –0.50 V. The proximity of this gate voltage to zero, combined with the near-linear variation of the current with the electric field exhibited for this condition (see Fig. 3c), suggests that it corresponds to the Dirac point. Indeed, as gate the voltage is swept away from this point in either direction, the overall current level increases monotonically, just as one would expect around the Dirac point. The behavior in Fig. 3b is very different, however, where we show the corresponding variation of current density with an electric field for the same device as in panel (a), in this case over the gate-voltage range of –5.00 V ≤ *V*_g_ ≤ –2.00 V. As the gate bias is reduced from –2.00 to –3.75 V, we see that the overall current level decreases rather than showing the increase that we might expect based on the trend from panel (a). At *V*_g_ ≤ –3.75 V, the current reaches another local minimum, where it once again exhibits a quasi-linear dependence on electric field (see Fig. 3c). With the further lowering of the gate voltage towards *V*_g_ = –5.00 V the current then again begins to increase, providing confirmation that the state at *V*_g_ = –3.75 V corresponds to a local minimum.

To clarify the details of the hot-carrier dynamics suggested in Fig. 3a, b, in panels (d)–(f) we plot detailed resistance contours for GhBN2 (at respective temperatures of 300, 77 and 3 K). In contrast to Fig. 2b, the contour of Fig. 3d exhibits two clear peaks; the first of these (near *V*_g_ = 0.5 V) is associated with the usual Dirac-point crossing, while the second is located on the hole side (near *V*_g_ = 3.7 V) of this peak. Although the hole-side feature is clearly visible at this relatively high temperature (300 K), there is no sign of any corresponding peak on the electron side. This is a common observation in many prior studies of linear transport in Moiré bands^7,9,11,12,14,15,17,18^, and is understood to result from the presence of a smaller Moiré gap for electrons than for holes^10^ (as we represent schematically in Fig. 1b). Both the main Dirac peak and the hole-related Moiré feature broaden as the electric field is increased, until, at the highest average field of 60 kV/cm, only the main Dirac feature remains visible.

**Fig. 4:**
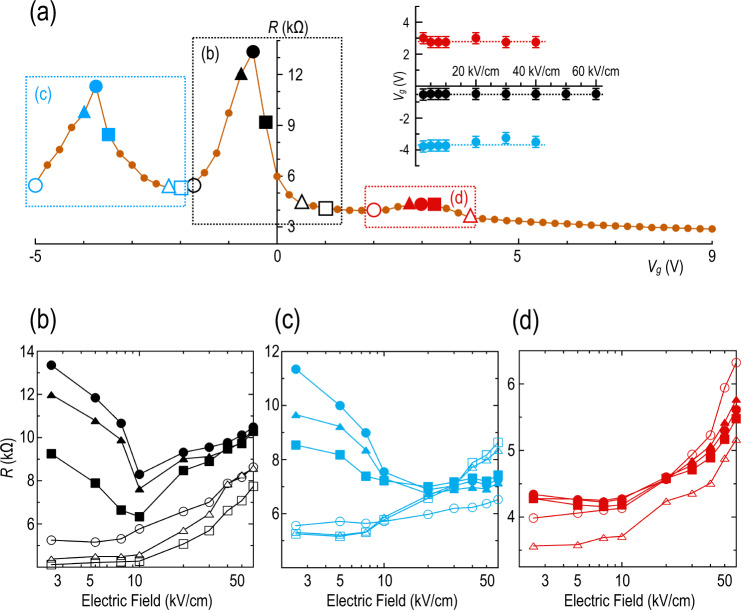
Metallic and semiconducting resistance states. **a** Variation of resistance of GhBN2 with gate voltage. The resistance here was determined by transient pulsing, performed at an average electric field of 2.5 kV/cm. **b–d** Variation of the transient resistance as a function of the average electric field, at gate voltages identified by the corresponding symbols in panel (**a**). The inset shows the position of the secondary (red and blue) and primary (black) Dirac peaks as a function of average electric-field strength at 3 K. The different colored symbols shown in this plot correspond to the peaks identified in Fig. 3f and the main panel of (**a**). Dotted lines are a guide to the eye. Error bars denote the experimental accuracy with which the different peaks can be identified from the data. Panel (**b**) shows the behavior observed near primary Dirac peak. Panel (**c**) shows the behavior observed near valence-miniband secondary peak. Panel (**d**) shows the behavior observed near conduction-miniband secondary peak. Data of panels **a**–**d** were all obtained at 3 K.

To reveal the influence of the Moiré spectrum of GhBN2 more prominently, in Fig. 3e, f we plot its resistance contours at respective temperatures of 77 and 3 K. These figures clearly show how the two original peaks seen at 300 K sharpen considerably with a reduction of temperature. In addition, in Fig. 3f a small but distinct peak can now be seen (near +2.9 V) on the electron side of the transfer curve. Referring to the behavior at 3 K, all three peaks broaden and diminish in amplitude with an increase of the electric field; while the secondary (electron and hole) structures are fully washed out by ~40 kV/cm, the main Dirac peak nonetheless remains visible at the highest average applied field of 60 kV/cm. In the inset to Fig. 4a, we show that the gate voltage position of each of the peaks does not change measurably with electric field. This indicates that application of the pulsed fields in these experiments does not induce significant “doping” shifts in the carrier system. From the estimated capacitance of the top gate, and the positions of the primary and secondary Dirac peaks, we estimate (see the “Methods” section) a twist angle of ~1° between the graphene and one of the h-BN layers. The absence of multiple peaks on the electron and hole sides of the primary Dirac point suggests^14,23^ that this (uncontrolled) Moiré structure arises from the coupling between the graphene and just one of the h-BN layers.

### Reentrant metallic and semiconducting states in the Moiré bands of graphene

The multi-peaked structure seen at low fields in Fig. 3f is generally understood^7^ to be associated with the opening of (inequivalent) gaps in the conduction and valence bands, and at the Dirac point itself (see Fig. 1b). Consequently, it is of interest to consider how the resulting hot-carrier dynamics is modified by the presence of this miniband structure. To address this question, in the different panels of Fig. 4, we plot the resistance of GhBN2 (at 3 K) as a function of the electric field and at a few distinct gate voltages. These voltages are identified in Fig. 4a, in which we plot the variation of *R*(*V*_g_) for GhBN2 (determined using a pulsed voltage of amplitude 250 mV, equivalent to a fairly low average electric field of 2.5 kV/cm). Referring to the behavior exhibited around the primary Dirac peak (near *V*_g_ = 0.5 V), the local resistance minima that flank this feature should correspond to a situation in which the Fermi level lies away from any minigap, being located instead within either the conduction or the valence miniband. Consequently, in Fig. 4b we observe that the resistance exhibits (see the data plotted as open symbols) behavior reminiscent of that found for ungapped graphene (Fig. 2c), increasing monotonically with increasing electric field. A more complicated behavior is found closer to the Dirac peak, however, where the Fermi level should lie in the minigap (of size Δ, see Fig. 1b) between the conduction and valence bands. Now, the resistance first decreases as the electric field is increased, before crossing over to the opposite trend at average fields beyond ~10 kV/cm (see the data plotted as filled symbols in panel (b)).

Next, we turn to the corresponding behavior exhibited in the vicinity of the hole-related peak, where the Fermi level should lie in the minigap Δ_h_ in Fig. 1b. In Fig. 4c we plot the variation of resistance with electric field at and around this feature. The resulting curves look very much like those of Fig. 4b; in the minima on either side of the peak the resistance increases in a “metallic” fashion with increasing electric field (open symbols). Near the secondary peak, on the other hand, we again observe (see filled symbols) a non-monotonic variation of resistance, with a semiconductor-like dependence below ~20–30 kV/cm that eventually evolves towards a metallic-like behavior at the highest electric fields.

Finally, we address the behavior observed near the secondary Dirac point in the conduction band. It is well established from both experiment and theory that the induced gap (Δ_*e*_) is much smaller here, typically exhibiting only a nascent character (while^7^ the secondary gap in the valence band is around 20–25 meV and the primary Dirac gap is ~35 meV). Consequently, when we plot (Fig. 4d) the variation of resistance with the electric field we observe only a weak non-monotonicity with the gate voltage tuned to this secondary peak. The resulting variation looks very much like that obtained on the flanks of the peak, where the resistance increases only slowly up to ~10 kV/cm, following which it begins to rise much more quickly.

To summarize our experimental findings, we have observed a cyclical evolution between metallic and semiconducting resistance variations as the Fermi level is moved through the graphene Moiré minibands. In the “semiconducting” state, the resistance decreases initially with increasing electric field, before crossing over to a metallic-like increase that onsets somewhere in the range of 10–30 kV/cm. These effects are most pronounced near the primary and the hole-related Dirac peaks, whose induced gaps are generally known to be the largest. At the much smaller electron peak, on the other hand, the difference between metallic and semiconducting variations cannot clearly be resolved.

### Theoretical analysis of hot-carrier transport in the Moiré bands of graphene

Motivated by the capacity of transient measurements to probe the intrinsic properties of small devices, while strongly suppressing extrinsic effects due to self-heating^36,37^, we provide insight into our experiment by using non-equilibrium Green’s function theory to formulate a model of hot-carrier transport in monolayer graphene^35^. The model describes transport in the presence of a spatially uniform electric field and of a parameterized energy gap (2Δ, representing the influence of the Moiré potential). Electron–phonon coupling is primarily considered to involve the optical phonons (with energy *ħω*_ph_ = 210 meV) that are known to dominate the high-field transport of graphene. The electrons and phonons are treated on an equal footing, accounting self-consistently for their self-energies within the dynamical mean-field approximation. To describe dissipation in the DC limit, we allow the electrons and phonons to decay into fermionic and phononic baths (at respective rates Γ and *τ*_p_^−1^), which are kept at the initial (equilibrium) substrate temperature (*T*_o_ in Fig. 1d). The essential components of this model are illustrated schematically in Fig. 1d, in which the fermion bath may be considered to represent the source and drain contacts. The rate *τ*_p_^−1^ represents the decay of non-equilibrium optical modes in the graphene to other phononic channels, including its own acoustic branches and the vibrational modes of the h-BN. The electron–phonon coupling term in the Hamiltonian is parametrized via the matrix element *g*_ep_ (with the corresponding quantity *g*_ep_^2^/*ħω*_ph_ representing the shift of electronic potential due to electron–phonon coupling) and accounts naturally for both the emission and absorption of phonons by hot carriers. The parameter *τ*_p_ may be viewed as representing the lifetime of non-equilibrium phonons, generated by emission from the hot carriers. In a situation in which all other parameters are held fixed, increase of *τ*_p_ will lead to both a rise in the effective temperature (*T*_ph_) of these phonons and to increased carrier scattering. We refer the reader to the “Methods” section and to the Supplementary Information for further details of the model.

**Fig. 5:**
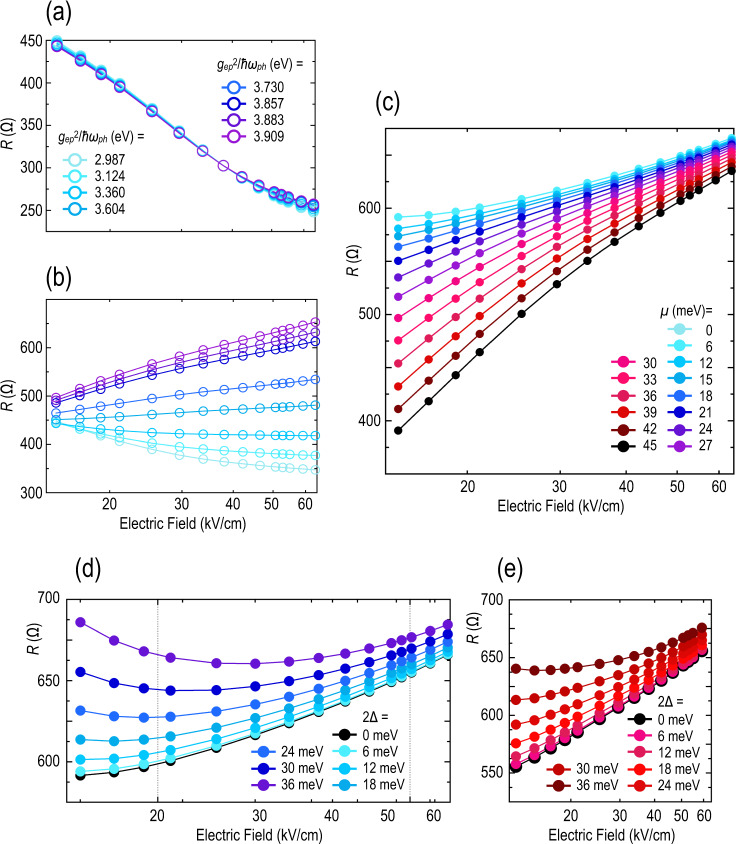
Calculated resistance under non-equilibrium for gapped and ungapped graphene. **a** Calculated variation of resistance as a function of average electric field for monolayer graphene (at *μ* = 30 meV). Calculations are performed assuming cold phonons and various values of the electron–phonon coupling strength *g*_ep_^2^/*ħω*_ph_ (indicated). **b** A similar calculation to that of panel (**a**), in which hot-phonon effects are accounted for. Symbols are the same as in panel (**a**). **c** Calculated variation of resistance as a function of average electric field for monolayer graphene and for various values of the chemical potential (*μ*, indicated). The calculations are for *g*_ep_^2^/*ħω*_ph_ = 3.909 eV and include hot-phonon effects. **d** Calculated variation of resistance as a function of average electric field for monolayer graphene (*μ* = 0) with an artificially induced gap of 2Δ (values indicted) around the Dirac point. **e** Similar plot to that of panel (**d**), but for chemical potential, *μ* = 18 meV. Other relevant parameters in panels **a**–**e** are as follows: *T*_o_ = 3.5 K, *ħω*_ph_ = 210 meV, *τ*_p_ = 4 ps & Γ = 4.5 meV.

To provide a comparison with the experimental results of Fig. 2, in Fig. 5a–c) we plot the calculated variation of resistance with an electric field for un-gapped graphene (i.e., for graphene with no Moiré-induced features). Panels (b) and (a) were obtained with and without hot-phonon effects, respectively, by fixing the chemical potential at 30 meV (in the conduction band) and varying the strength (*g*_ep_^2^/*ħω*_ph_) of the electron–phonon coupling. In the cold-phonon model of Fig. 5a, the resistance decreases monotonically with increasing electric field, over the entire range of considered coupling strength. Since hot-phonon effects are (artificially) suppressed here, this behavior results mainly from the influence of Landau–Zener tunneling at non-zero fields; by exciting carriers from the valence to the conduction band, this mechanism increases the number of carriers available for conduction^35^ and so leads to the decrease of resistance with electric field shown in panel (a). The situation is very different in Fig. 5b where, for *g*_ep_^2^/*ħω*_ph_ > 3.7 eV, the resistance now increases with the electric field. Basically, what is happening here is that the increased electron–phonon coupling is allowing hot-phonon effects to dominate over Landau–Zener tunneling, resulting in a very different variation of resistance. In Fig. 5c, in which the role of hot phonons is also included, we set the electron–phonon coupling at *g*_ep_^2^/*ħω*_ph_ = 3.91 eV and calculate the variation of resistance with an electric field for various initial values of *μ* in the conduction band. These results capture the effect of varying the gate voltage in the experiment and demonstrate that a proper treatment of hot-phonon effects, and their competition with Landau–Zener tunneling, is needed to accurately describe transport in graphene under non-equilibrium. At higher electric fields (>30 kV/cm) the calculations show a good correspondence with those of Fig. 2c. (At lower fields the experimental and theoretical trends differ, with the resistance in the former case saturating as the field is lowered towards zero; this may reflect the influence of other scattering mechanisms – such as impurities – not included in our model.) At the Dirac point (*μ* = 0), the resistance varies only slowly with an electric field, reflecting the weak influence of electron-phonon effects in the presence of the small Fermi surface. As we move away from the Dirac point, however, the resulting increase in the Fermi surface allows non-equilibrium hot phonons to enhance electron scattering, leading to an increased variation of the resistance. This behavior not only resembles that found for un-gapped graphene but is also like that obtained away from the Moiré resistance peaks of Fig. 4 (see panels b–d, data plotted with open symbols).

Moving to the behavior expected when the Fermi level lies within a Moiré gap, in Fig. 5d we plot the calculated variation of resistance with an electric field for graphene with an artificially imposed gap (2Δ, centered around the Dirac point). As the size of this gap is increased the resistance develops an increasingly non-monotonic nature, first decreasing with increasing electric field before crossing over to an increasing resistance at higher fields. This non-monotonic variation, too, can be understood in terms of the competition of Landau–Zener tunneling and hot-phonon action. The field-assisted tunneling is the predominant mechanism at lower electric fields, initially leading to an increase in current (i.e., to a decrease in resistance) as the field is raised. At higher fields, however, where the presence of the gap has now essentially been overcome by the Landau–Zener excitation, increased carrier scattering by hot phonons leads instead to a metallic-like increase of the resistance. The overall variation observed with a non-zero gap in Fig. 5d is clearly reminiscent of that found at the Moiré resistance peaks of Fig. 4 (see panels b–d, data plotted with filled symbols).

While the data of Fig. 5d were obtained for a configuration in which the Fermi level was set at mid-gap (*μ* = 0), similar results are also obtained away from this condition. The results of Fig. 5e, for example, assume *μ* = 18 meV and correspond to a Fermi level that lies well within the conduction band. When 2Δ < 36 meV, this situation mimics the behavior away from either the primary or secondary Dirac peaks (data with open symbols in Fig. 5b–d), consistent with which we find that the resistance is metallic and increases only monotonically with the electric field. For 2Δ = 36 meV, in contrast, the influence of the gap begins to be manifested and the resistance develops the non-monotonic character that reflects the competition of Landau–Zener tunneling and scattering by hot phonons.

## Discussion

**Fig. 6:**
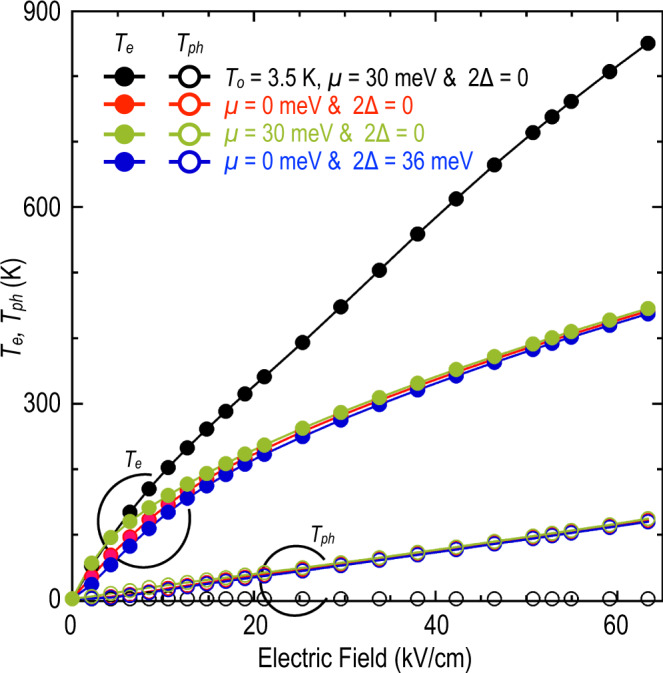
Electron and phonon temperatures under non-equilibrium. Variation of electron (filled symbols) and lattice (open symbols) temperatures as a function of the electric field, calculated for various scenarios within our model. Black data points show *T*_e_ and *T*_ph_ for a “cold-phonon” model, in which we assume that the phonon system remains fixed at its initial (zero-field) value of 3.5 K. Other colors correspond to different combinations of *μ* and 2Δ, as indicated in the legend (*ħω*_ph_ = 210 meV, *τ*_p_ = 4 ps, and Γ = 4.5 meV).

As is common in discussions of non-equilibrium carrier phenomena in semiconductors^33,39^, the theoretical model that we have developed describes high-field transport in graphene by self-consistently accounting for the induced variations in both the electron (*T*_e_) and lattice (*T*_ph_) temperatures. These temperatures are deduced in standard fashion, from the form of the electron and phonon distribution functions under non-equilibrium (as described in Eqs. (S18)–(S20) of the Supplementary Information). The extent to which these temperatures are expected to vary is highlighted in Fig. 6, where we compare the predictions for two scenarios. The first of these (open and filled black symbols) involves the assumption of a “cold-lattice”^35^, in which we artificially impose an instantaneous phonon decay (*τ*_p_ = 0) to hold *T*_ph_ at its initial (equilibrium) value (*T*_ph_ = *T*_o_ = 3.5 K). Here we see that the electron temperature increases almost linearly with an electric field, approaching 900 K at the highest fields. Similarly high temperatures have been obtained previously in Monte-Carlo simulations of high-field transport in graphene^40^. The near-linear variation of *T*_e_ with an electric field is reasonable for the cold-phonon scenario, for which the supplied electrical power (proportional to the square of the electric field) is dissipated in the carrier system alone. Noting that the energy of a degenerate Fermi gas (at low temperatures) varies as *T*_e_^2^, we might then expect a simple linear dependence of *T*_e_ with the electric field. This model is clearly unphysical, however, as we have seen already (recall Fig. 5a) that the cold-phonon assumption fails to correctly reproduce the experimental resistance trends. This instead requires a self-consistent treatment of the electron–phonon coupling, in which both *T*_e_ and *T*_ph_ are allowed to vary with the electric field. This is highlighted by the colored (red, green, and blue) data points of Fig. 6. The lattice temperature now increases to more than 100 K at 65 kV/cm, while the inclusion of lattice heating means that the carrier temperature increases more slowly than in the cold-phonon case, reaching only 450 K for the highest electric fields. For fields larger than 20 kV/cm, both *T*_e_ and *T*_ph_ appear insensitive to variation of the Fermi level or to whether the graphene is gapped or un-gapped. We attribute this to the fact that transport is dominated by scattering from optical phonons at high fields, independent of the details of low-field transport.

In very recent work^41^, novel signatures of the so-called “Dirac fluid” have been studied in graphene superlattices, realized with precise control of the interlayer twist angle. The emergence of controlled mini-gaps in the bandstructure of these systems enables a situation in which the drift velocity of driven carriers can be comparable to the Fermi velocity, leading to a transition to a state in which the filled bands begin to play an essential role in transport. The critical phenomena observed under those conditions were achieved at current densities some two orders of magnitude lower than those reached here, as a result of which the influence of phonon-related effects was not considered. This should therefore be contrasted with our study, performed deep in the dissipative regime, in which we have seen how it is essential to account for the non-equilibrium nature of both the carrier and phonon populations.

In this study, we have demonstrated the presence of re-entrant metallic and semiconducting states in a Moiré-gapped h-BN/graphene/h-BN heterostructure. The semiconducting states are observed when the chemical potential is swept through the commensurability induced gaps in the graphene spectrum, while metallic behavior is obtained when this level is located within the minibands bound by these gaps. To account for the experimentally observed behavior, we have developed a model of non-equilibrium transport that accounts self-consistently for the coupling of the carrier and lattice systems. The picture that emerges from this model is one in which the experimentally observed resistance variations can be attributed to a competition between electrically driven inter-band (Landau–Zener) tunneling and nonequilibrium electron–phonon coupling. The latter dominates the metallic resistance variations, found for ungapped graphene or when the Fermi level lies within a Moiré miniband. With the Fermi level in a minigap, however, Landau–Zener tunneling dominates at low fields before hot-phonon effects take over at higher fields. It is this competition that results in the nonmonotonic resistance variations characteristic of the semiconducting states. Overall, our results provide important insight into hot-carrier dynamics in graphene/h-BN heterostructures and the impact of hot phonons on their electrical performance.

## Methods

### Graphene/h-BN heterostructure fabrication

The van der Waals heterostructures studied here were assembled by the PC (6% poly bisphenol A carbonate dissolved in chloroform)/poly dimethylpolysiloxane- (PDMS) based dry-transfer method^34,42^. In this approach, graphene flakes and h-BN crystals were exfoliated onto SiO_2_/Si (300 nm/0.5 mm) chips from bulk crystals. The material used for device fabrication was carefully chosen by means of optical microscopy, Raman spectroscopy, and atomic force microscopy (AFM, see Sections S2 and S3 of the Supplementary Information). To facilitate high-speed electrical pulsing of the devices, they were fabricated with a simple transistor geometry comprising source and drain contacts to the graphene layer, and an isolated top gate whose dielectric was provided by the upper h-BN layer^38^ and a 20-nm HfO_2_ film. The two contacts to the graphene were formed by the one-dimensional edge contact technique^34,42^, using a combination of electron-beam lithography (EBL), reactive-ion etching (in a gas mixture of CHF_3_ and O_2_), and electron-beam evaporation (Cr/Pd/Au: 3-/15-/80-nm). In this two-step process, the edge contacts were first fabricated using a high-resolution EBL exposure. Next, the large coplanar waveguides were fabricated in a second EBL step, in which a system of on-chip alignment markers was used to accurately overlap the waveguides with the two edge contacts. The extended metallized contacts formed in this way are denoted by white dotted lines in the lower inset to Fig. 2a. As can be seen here, the input and output signal lines extend as far as their respective edges of the top-gate, meaning that the active graphene channel is defined immediately underneath that gate. Here we report results from the study of two separate monolayer-graphene devices that we refer to as GhBN1 and GhBN2. In both devices, the gap in the coplanar-waveguide signal line, representing the source–drain separation, was 0.5 μm and the width of this line was 7.2 μm. The waveguide structure was positioned relative to the graphene sheet in such a manner that the channel width of the resulting transistor corresponded closely (to better than 10%) to that of the signal line. The twist angle between the different layers was not controlled in our study, implying that induced Moiré structure could only be achieved in an unintentional fashion. Here we, therefore, report the results from studies of two devices: one (GhBN2) in which clear signatures of Moiré minibands were observed, and a second (GhBN1) in which such features were absent. The thickness of the upper (lower) h-BN layer in these devices was 152 nm (161 nm) in GhBN1 and 92 nm (154 nm) in GhBN2.

### High-speed transient measurements

Pulsed current−voltage characteristics of the devices were measured in a repetitive-pulsing scheme^36,43,44^. The measurements were made in a custom-designed setup with full (50-Ω) impedance matching. The device chip was mounted on an FR-4 laminate board, allowing the signal lines of its coplanar waveguide to be connected to semirigid coaxial cables. Single-shot or repetitive pulses were applied with an AVTECH generator, providing a maximum pulse amplitude of 10 V and a repetition frequency that was set at 10 kHz to avoid the influence of self-heating^38^. The pulse duration could be varied from a few ns to a microsecond and was chosen here to be 60 ns; for this optimal duration, the steady-state current reached with the pulse applied could be accurately identified yet did not differ noticeably from that obtained using shorter pulses (down to a few nanoseconds, see Section S1 of the Supplementary Information for further details of the pulsed-measurement scheme). Resulting output pulses were then captured by a fast oscilloscope (a Tektronix CSA8000B digital-sampling oscilloscope with 50 GHz bandwidth or a Keysight MSOX6004A mixed-signal oscilloscope with 6 GHz bandwidth).

### Determination of twist angle from primary and secondary Dirac peaks

In GhBN2, the presence of just one secondary peak due to each of the valence and conduction miniband indicates^14^ that the Moiré states result from the twist angle between the graphene and just one of the h-BN layers (we do not know which). To estimate the twist angle between these two layers, we use the (gate-voltage) separation between the two secondary peaks and the primary Dirac peak (Δ*V*_g_ ~ 3.2 V, see inset to Fig. 4a), and the top-gate capacitance of 3.1 × 10^−4^ F m^−2^, to determine an associated carrier doping change of *n* = 6.24 × 10^15^ m^−2^. Using the relation:^9^

1 $$\lambda=\frac{\left(1+\delta \right)a}{\sqrt{2\left(1+\delta \right)\left[1-{{\cos }}\theta \right]+{\delta }^{2}}}$$

where *δ* = 0.017 expresses the lattice mismatch between graphene and h-BN, *a* = 0.246 nm is the lattice constant of graphene (the distance between nearest C atoms of the same triangular sub-lattice), *λ* is the twist angle between the layers, and *θ* is defined from the carrier concentration at the secondary peaks according to $n=\sqrt{8/3{\lambda }^{2}}$, we estimate a corresponding angle of ~1°.

### Theoretical methods

We model transport in monolayer graphene by using the non-equilibrium Green’s function method, placing electrons and phonons on an equal footing by self-consistently computing their self-energies. The graphene layer, with on-site optical phonons of energy *ħω*_ph_ = 210 meV, is modeled on an infinite honeycomb tight-binding lattice subject to the electrostatic potential *V*(**r**) = −*eEx* (where the electric field *E* is assumed to be uniform and directed along the *x* direction). The electron-phonon self-energies are then approximated within the dynamical mean-field theory^45^. The electron and phonon baths are added by means of dissipative self-energies, facilitating the steady-state DC limit^46^ [which we impose on the Green’s function via *G*(**r** + **a**, *ω*_ph_) = *G*(**r**, *ω*_ph_ + e**E**・**a**/*ħ*) when the position **r** is displaced by lattice vector **a**]. Dissipation is controlled by means of the electron dissipation rate Γand the hot-phonon decay rate *τ*_p_^−1^ into Ohmic baths. To model transport in graphene with a gap induced by the Moiré effect, we add the energy displacement +Δ and –Δ, respectively, to the A- and B-sublattices of the honeycomb crystal. For further details on the theoretical procedures, we refer the reader to Section S4 of the Supplementary Information.

### Reporting summary

Further information on research design is available in the Nature Portfolio Reporting Summary linked to this article.

## Supplementary information

Supplementary Information

Reporting Summary

## Data Availability

Source data are provided with this paper.

## Source data

Source Data

## Code availability

The computational code required to reproduce the theoretical calculations presented in this paper is available from the corresponding authors upon reasonable request.
