# Supplementary material for: Signatures of hot carriers and hot phonons in the re-entrant metallic and semiconducting states of Moiré-gapped graphene
Source: Nat Commun. 2023 Mar 17;14:1507. doi: 10.1038/s41467-023-37292-4 (PMC10023744; doi:10.1038/s41467-023-37292-4)
Supplement: Supplementary file 1 — Supplementary Information [file 41467_2023_37292_MOESM1_ESM.pdf]

SUPPLEMENTARY INFORMATION

Hot Carriers, Hot Phonons and Re-Entrant Metallic and Semiconducting States in Moiré-Gapped Graphene

Jubin Nathawat,[1] Ishiaka Mansaray,[2] Kohei Sakanashi,[3] Naoto Wada,[3] Michael D. Randle,[1] Shenchu Yin,[1] Keke He,[1] Nargess Arabchigavkani,[1] Ripudaman Dixit,[1] Bilal Barut,[3] Miao Zhao,[4] Harihara Ramamoorthy,[5] Ratchanok Somphonsane,[5] Gil-Ho Kim,[6] Kenji Watanabe,[7] Takashi Taniguchi,[7] Nobuyuki Aoki,[3] Jong E. Han,[2,*] and Jonathan P. Bird[1,†]

[1]*Department of Electrical Engineering, University at Buffalo, the State University of New York, Buffalo, NY 14260-1900, USA*

[2]*Department of Physics, University at Buffalo, the State University of New York, Buffalo, NY 14260-1500, USA*

[3]*Department of Materials Science, Chiba University, Inage-ku, Chiba 263-8522, Japan*

[4]*High-Frequency High-Voltage Device and Integrated Circuits Center, Institute of Microelectron-ics of Chinese Academy of Sciences, 3 Beitucheng West Road, Chaoyang District, Beijing 100029, PR China*

[5]*Department of Physics, Faculty of Science, King Mongkut's Institute of Technology Ladkrabang, Bangkok, 10520 Thailand*

[6]*School of Electronic and Electrical Engineering and Sungkyunkwan Advanced Institute of Nanotechnology (SAINT), Sungkyunkwan University, Suwon 16419, Korea*

[7]*Advanced Materials Laboratory, National Institute for Materials Science, Tsukuba 305-0044, Japan*

[*] jonghan@buffalo.edu

[†] jbird@buffalo.edu

S1. TRANSIENT-MEASUREMENT TECHNIQUE

In recent years, we have made extensive use of the transient-measurement technique to study the dynamics of hot carriers in a variety of different systems (see Refs. [1–6]). A detailed description of this approach, as applied to the measurement of two-dimensional semiconductors such as graphene, can be found in Ref. [2]. The basic details of this scheme are described in the Methods section of the main paper. Here we address two important aspects of the transient approach, namely: (1) the way we identify the appropriate pulse duration for use in our studies, and (2) the method we use to determine the transient current-field characteristics – and transient resistance (R) – of the devices.

S1.1. Selecting the Transient Pulse Duration

A key objective in many transient measurements (including those of interest here) is to probe the electrical characteristics of some system or device of interest, by subjecting it to pulses that are sufficiently short to minimize the effects of self-heating (*i.e.*, heating of those parts of the device – typically the underlying substrate – other than the conducting channel itself). In our experiments, we achieve this objective by reducing the pulse duration to the nanosecond range, where the transient current observed during the steady-state portion of the pulse is found to be time independent. This behavior is illustrated in Fig. S1, in the left panel of which we show transient pulses of nominal amplitude 5.00 V, and duration ranging from 4 ns to 60 ns, as generated by our power supply. In the right-hand panel of the figure, we show the corresponding transient voltage ($V_{\text{out}}(t)$) measured at the 50-Ω input of our oscilloscope, with these respective pulses applied to one of our devices. It is clear from a comparison of these two panels that, for the range of pulse durations considered here, the transient current [equal to $V_{\text{out}}(t)/50$] closely follows the form of the applied voltage pulses. Most notably, over the portion of the applied transient corresponding to its steady-state output we see that the transient current through the device is similarly time- (or pulse-duration) independent. In this way, we confirm that our measurements are performed in a limit in which the influence of extrinsic self-heating is suppressed. Noting the behavior shown in Fig. S1, we set the pulse duration in this study to be 60 ns; while this remains in a limit of weak self-heating, it is sufficient to allow us to clearly identify the steady-state value of the transient current.

S1.2. Parameter Extraction from the Pulsed Measurements

In the different figures of the main paper, we discuss a number of parameters that are extracted from the measured current transients. The transient current-field characteristics plotted in Figs. 2(a) & 3(a) – 3(c), for example, were obtained in the following way. The average electric field of each data point was calculated by first determining the steady-state value of the voltage drop across the device, as the difference between the input transient and the corresponding variation of the output voltage measured by the oscilloscope ($V_{\text{in}} - V_{\text{out}}$, as identified by the dotted lines in the respective panels of Fig. S1). By dividing this voltage drop by the channel length (0.5 μm), we thus arrive at the required electric field. The corresponding current density (J) at this field was

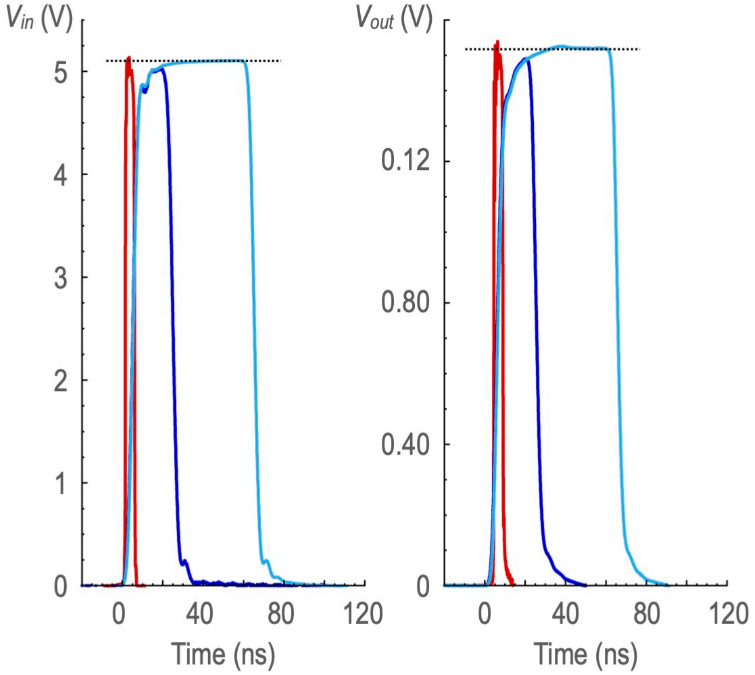

Fig. S1. The left panel shows applied voltage pulses of peak amplitude 5.00 V, and of various steady-state duration (4 ns, 20 ns and 60 ns), as generated by our pulsed source. The waveforms were recorded by feeding the output of the pulse generator directly into the 50-Ω input of our oscilloscope. The right panel shows the resulting variations of the transient voltage, measured at the same 50-Ω input, after the pulses plotted in the left panel were applied to one of our devices. Dotted lines in both panels denote steady-state values of the respective pulsed waveforms. The variation of the transient current, represented in the right panel, closely follows the form of the applied transient voltage on the left. Dotted lines in both panels identify steady-state values of V_{in} and V_{out}, used to calculate the transient current-voltage curves presented in the main paper.

then obtained by converting the steady-state value of V_{out} by the 50-Ω input impedance of the oscilloscope and dividing this result by the channel width (7.2 μm).

The resistance contours of Figs. 2(b) & 3(d) – 3(f) plot the variation of measured resistance (R) as a function of electric field and gate voltage. The contours were constructed by determining the steady-state voltage drop across the device as described above and dividing this by the corresponding steady current value ($V_{\mathrm{out}}/50$). This procedure was repeated for each condition, as the pulse amplitude and the gate voltage were incremented. The resistance defined in this manner is therefore an absolute one, rather than a differential quantity.

S2. RAMAN CHARACTERIZATION OF FABRICATED SAMPLES

Raman spectra of the graphene monolayers were measured at room temperature under ambient atmosphere. Results are plotted in Fig. S2. The large ratio (> 2) of the 2D-to-G peak amplitudes is consistent with the presence of monolayer graphene of good

quality.

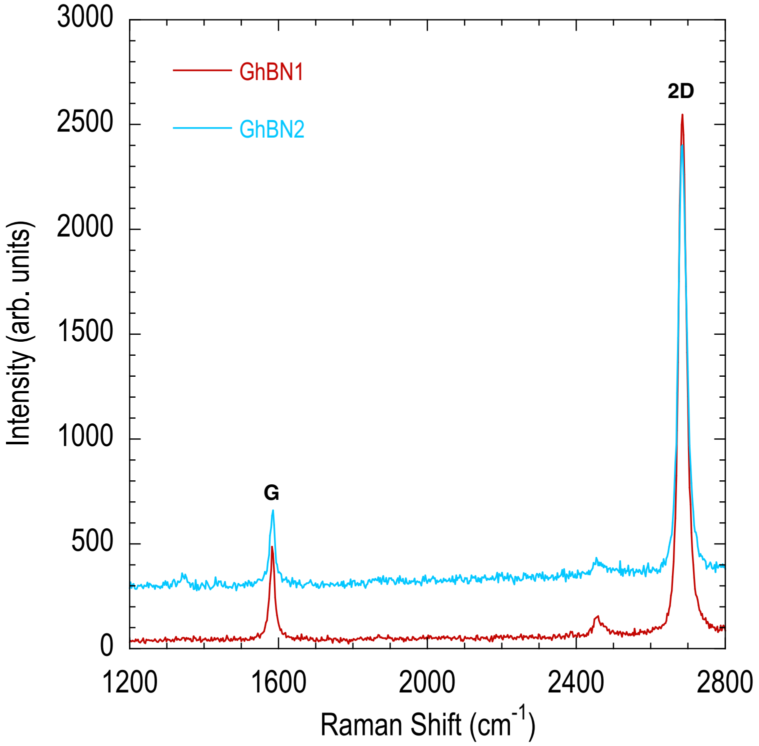

Fig. S2. Raman spectra of the graphene monolayers used to realize devices GhBN1 and GhBN2. For GhBN2, a non-zero background signal is present but is clearly distinguished from the relevant Raman peaks (G and 2D). Measurements made under ambient conditions before full encapsulation of the graphene in h-BN.

S3. ATOMIC FORCE MICROSCOPY OF DEVICE LAYER STRUCTURE

Thickness of the upper and lower layers of h-BN, and of the upper HfO_2 gate dielectric could be determined by using atomic force microscopy to read off these values. An illustration of the manner in which this could be done is provided in Fig. S3.

S4. THEORY: MODEL AND GREEN'S FUNCTION FORMALISM

Model: The Hamiltonian consists of electron and phonon parts as [7]

$$H = H_{\rm el} + H_{\rm ph} + H_{\rm ep} + H_{\rm diss}, \tag{S1}$$

with the electron part $H_{\rm el}$ modeled on a tight-binding lattice with electrostatic potential $V(\mathbf{r}) = -e\mathbf{E}\cdot\mathbf{r}$ on the site $\mathbf{r}$ of an infinite honeycomb lattice, the phonon part $H_{\rm ph}$ with Einstein phonons of energy $\hbar\omega_{\rm ph}$, the electron-phonon (el-ph) coupling $H_{\rm ep}$ and the dissipation mechanism for both electrons and phonons being contained in $H_{\rm diss}$. The electron part is written as

$$H_{\rm el} = -t\sum_{<\mathbf{r},\mathbf{r}'>} d^\dagger_{\mathbf{r}} d_{\mathbf{r}'} - \sum_{\mathbf{r}}(e\mathbf{E}\cdot\mathbf{r})d^\dagger_{\mathbf{r}} d_{\mathbf{r}}, \tag{S2}$$

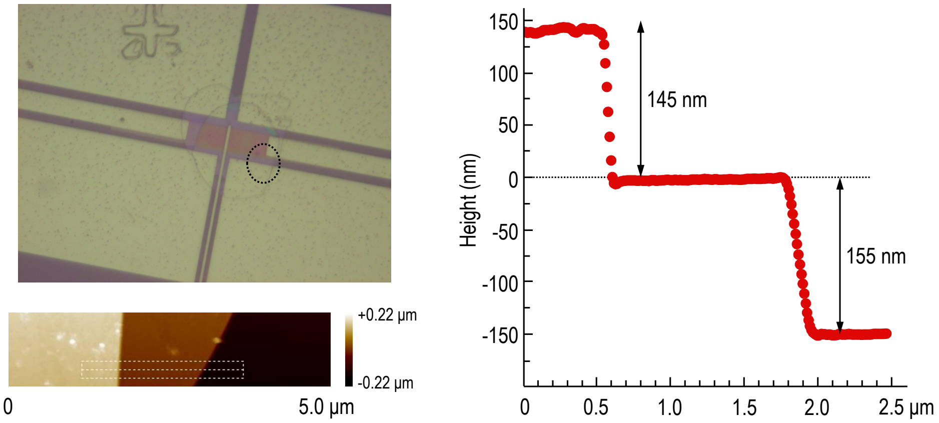

Fig. S3. AFM inspection of a similar device to those studied here. The upper-left image in an optical micrograph, in which the black dotted line encircles an area of interest. An AFM scan around this region, showing the transition from the SiO_2 substrate (black), to the top surface of the lower h-BN layer (dark brown) and, finally, to the top surface of the upper h-BN layer (light brown), is shown bottom left. The lineplot on the right shows a height profile along the white dashed line in the lower-left inset. This figure allows the thickness of the two h-BN encapsulation layers to be determined (as indicated).

with the electron creation (annihilation) operators $d^\dagger_{\mathbf{r}}$ ($d_{\mathbf{r}}$) on site $\mathbf{r}$. Here, we suppress the summation over the electron spin and take spin degeneracy in the calculations. The lattice summation is over the nearest neighbors $<\mathbf{r},\mathbf{r}'>$ of the infinite honeycomb lattice, with the tight-binding parameter t. Throughout this work we set $t = 3$ eV. We define the electric-field (E) direction as along the x-direction. The phonon part consists of Einstein phonons of energy $\hbar\omega_{\rm ph}$ on each site as $H_{\rm ph} = \frac{1}{2}\sum_{\mathbf{r}}(p^2_{\mathbf{r}} + \omega^2_{\rm ph}\varphi^2_{\mathbf{r}})$ with the phonon field $\varphi_{\mathbf{r}}$ defined on each site and its conjugate momentum $p_{\mathbf{r}}$. The electron-phonon coupling part reads

$$H_{\rm ep} = (2\omega_{\rm ph}/\hbar)^{1/2} g_{\rm ep} \sum_{\rm r} \varphi_{\mathbf{r}} d^\dagger_{\mathbf{r}} d_{\mathbf{r}} \qquad \text{(S3)}$$

with the coupling constant $g_{\rm ep}$. Here, the electron couples to the phonon amplitude $\varphi_{\rm r} = (\hbar/2\omega_{\rm ph})^{1/2}(a^\dagger_{\rm r} + a_{\rm r})$ with the superposition of emission and absorption of phonon quanta via $a^\dagger_{\rm r}$ and $a_{\rm r}$. The factor $(2\omega_{\rm ph}/\hbar)^{1/2}$ inserted in Eq. (S3) to keep the dimension of $g_{\rm ep}$ as energy. Energetic electrons accelerated by the electric field dump the excess energy to the phonon system via the inelastic process by $H_{\rm ep}$. We will use the simplified unit $\hbar = e = 1$ from now on.

The dissipation $H_{\rm diss}$ has two parts, for electrons and phonons, respectively, as $H_{\rm diss} = H_{\rm diss,el} + H_{\rm diss,ph}$. The dissipation into fermion baths can be understood as particle exchange between hot and cold electrons in the sample and substrate, respectively. We model the fermion bath as a continuum of fermion states, with each state being labeled as k, coupled to each site independently [7] as $H_{\rm diss,el} = \sum_{\mathbf{r}} H_{\rm diss,el}(\mathbf{r})$ with

$$H_{\rm diss,el}(\mathbf{r}) = \sum_k \left[(\epsilon_k - \mathbf{E}\cdot\mathbf{r})\, c^\dagger_{\mathbf{r}}(k) c_{\mathbf{r}}(k) + \frac{\gamma}{\sqrt{N}} d^\dagger_{\mathbf{r}} c_{\mathbf{r}}(k) + H.C. \right]. \qquad \text{(S4)}$$

Here, $c^\dagger_{\mathbf{r}}(k)$ ($c_{\mathbf{r}}(k)$) is the creation (annihilation) operator of bath fermions of continuum index k defined at each site $\mathbf{r}$, ϵ_k the dispersion relation, γ the overlap to the main orbital $d^\dagger_{\mathbf{r}}$ ($d_{\mathbf{r}}$), and N the normalization factor against the length of the bath chain. We assume a structureless model for the density of states of infinite bandwidth with the energy-independent hybridization Γ as

$$\frac{\gamma^2}{N}\sum_k \delta(\omega - \epsilon_k) \equiv \Gamma. \tag{S5}$$

The dissipation of the phonons can be formulated similarly, now with coupling to an Ohmic bath at each site. With $H_{\text{diss,ph}} = \sum_{\mathbf{r}} H_{\text{diss,ph}}(\mathbf{r})$

$$H_{\text{diss,ph}}(\mathbf{r}) = \sum_k \left[\frac{1}{2}\left(q^2_{\mathbf{r}}(k) + \nu_k^2 x^2_{\mathbf{r}}(k)\right) + \frac{\alpha_k}{\sqrt{N}} x_{\mathbf{r}}(k)\varphi_{\mathbf{r}} + H.C.\right]. \tag{S6}$$

Here, $x_{\mathbf{r}}(k)$ is the amplitude of the bath particle with the continuum index k and $q_{\mathbf{r}}(k)$ is its conjugate momentum, ν_k is the dispersion relation, and α_k is the overlap to the phonon field on the main lattice. In the Ohmic limit, we define the decay rate of the phonons into the bath to be

$$\frac{1}{N}\sum_k \frac{\alpha_k^2}{\nu_k^2}\delta(\omega - \nu_k) \equiv \tau_P^{-1}\omega, \tag{S7}$$

with the phonon life-time parameter τ_P.

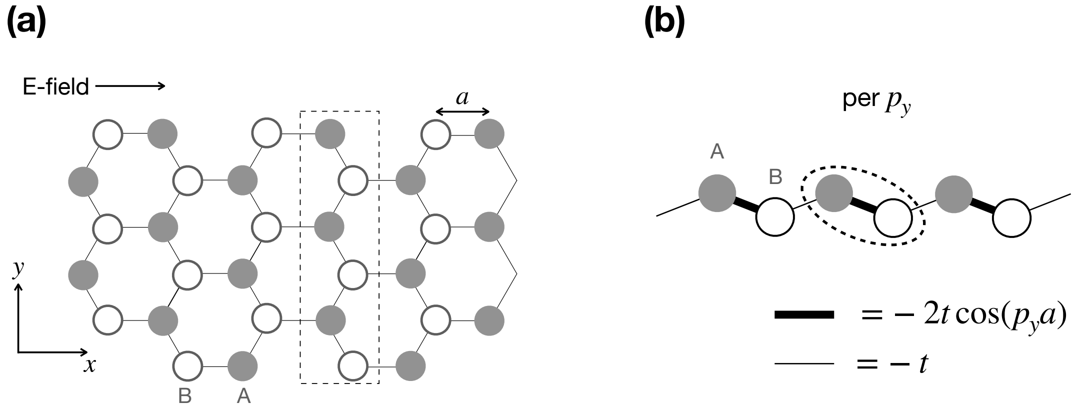

Fig. S4. Graphene lattice. (a) We construct the whole lattice in terms of the 'zigzag' cell (dashed rectangular box) that is stacked along the field direction. Inside the cell, there is translation invariance along the y-direction. (b) For each wave-vector p_y along the y-direction, the effective lattice can be viewed as a two-atom chain with the intra- and inter-chain coupling as shown.

Deconstruction of lattice: In a uniform electric field, translational symmetry for the parallel and perpendicular directions is considered differently. In the direction of the

field, translational symmetry is recovered in the (site-diagonal) electron Green's function if we shift the energy by the amount of the electrostatic potential,

$$G^{R,<}(\mathbf{r}+\mathbf{R},\omega) = G^{R,<}(\mathbf{r},\omega+\mathbf{E}\cdot\mathbf{R}), \tag{S8}$$

with the lattice displacement $\mathbf{R}$. In the direction perpendicular to the field, true translational invariance holds. Due to this symmetry, we deconstruct the lattice as shown in Fig. S4, with the rectangular cells inside of which we can use the plane-wave basis along the y-direction with the wave-vector p_y to partially diagonalize the basis as

$$d^\dagger_{\ell s}(p_y) = \frac{1}{\sqrt{N_y}}{\sum_{\mathbf{r}}}' e^{ip_y y} d^\dagger_{\mathbf{r}}, \tag{S9}$$

where N_y is for the normalization, ℓ the cell index, $s = A, B$ for the sublattice index, and the summation is over the sites in the box. The intra-chain tight-binding Hamiltonian becomes

$$H_{\mathrm{TB},\perp}(p_y) = -2t\cos(p_y a)\sum_\ell [d^\dagger_{\ell A}(p_y)d_{\ell B}(p_y) + H.C.] \tag{S10}$$

and the inter-chain coupling is

$$H_{\mathrm{TB},\parallel}(p_y) = -t\sum_\ell [d^\dagger_{\ell+1,A}(p_y)d_{\ell B}(p_y) + H.C.]. \tag{S11}$$

Hopping in $H_{\mathrm{TB},\perp}(p_y)$ and $H_{\mathrm{TB},\parallel}(p_y)$ is represented by the thick and thin lines, respectively, in Fig. S4(b). With the Ansatz of local self-energy in the dynamical mean-field theory (DMFT [9]), the Dyson equation for the nonequilibrium lattice can be constructed by using the iteration technique [8] along the effective chain per p_y [7]. The full local Green's functions are calculated by summing p_y over the Brillouin zone of p_y.

Green's function technique:

Within the Keldysh Green's function technique, the effect of a dissipative bath can be easily incorporated in the (non-interacting) self-energy as

$$\Sigma_0^R(\mathbf{r},\omega) = -i\Gamma \text{ and } \Sigma_0^<(\mathbf{r},\omega) = 2i\Gamma f_0(\omega+\mathbf{E}\cdot\mathbf{r}), \tag{S12}$$

for the retarded and lesser Green's functions for electrons, respectively. $f_0(\omega) = [e^{\omega/k_B T_b}+1]^{-1}$ is the Fermi-Dirac function at the bath temperature T_b (k_B = Boltzmann constant). Phonon dissipation is similarly described within the dissipative phonon self-energy as

$$\Pi_0^R(\omega) = -2i\tau_P^{-1}\omega \text{ and } \Pi_0^<(\omega) = -4i\tau_P^{-1}\omega n_0(\omega), \tag{S13}$$

with the Bose-Einstein function $n_0(\omega) = [e^{\omega/k_B T_b}-1]^{-1}$. Note that the electric field does not couple directly to the charge-neutral phonons and the phonon Green's functions are translationally invariant.

We solve the electron-phonon problem by mutually consistent interacting self-energies to electrons and phonons through iteration. The approximation is that we consider the perturbation up to second-order of the el-ph coupling g_{ep}. The electron self-energy by the el-ph coupling is given as

$$\Sigma^{\lessgtr}_{\mathrm{ep}}(\mathbf{r},\omega) = 2i\omega_{\mathrm{ph}}g^2_{\mathrm{ep}}\int\frac{d\omega'}{2\pi}\mathcal{G}^{\lessgtr}(\mathbf{r},\omega-\omega')D^{\lessgtr}(\omega'), \tag{S14}$$

where $\mathcal{G}^{\lessgtr}(\mathbf{r},\omega)$ is the electronic *impurity* Green's function in the DMFT formalism [9] and $D^{\lessgtr}(\omega)$ phonon's on-site Green's function. The phonon self-energy is similarly defined as

$$\Pi^{\lessgtr}_{\rm ep}(\omega) = -4i\omega_{\rm ph}g^2_{\rm ep}\int\frac{d\omega'}{2\pi}G^{\lessgtr}(\mathbf{r},\omega+\omega')G^{\gtrless}(\mathbf{r},\omega'), \tag{S15}$$

with the full electron Green's function $G^{\gtrless}(\mathbf{r},\omega)$. The pre-factor 4 is due to the spin degeneracy. The retarded and advanced self-energies are computed according to the standard definition [9]. Here, we use the convention that the phonon Green's function is defined with respect to the field variable, eg., $D^>(t) = -i\langle\varphi_{\mathbf{r}}(t)\varphi_{\mathbf{r}}(0)\rangle$.

Once the self-energies are computed, we construct the DMFT impurity Green's function $\mathcal{G}(\omega)$. Due to the broken spatial symmetry, the equilibrium version with the momentum summation is not applicable. The modified Dyson's equation becomes for the site $\mathbf{r}=0$,

$$\mathcal{G}^R(\omega) = [I + G^R(\omega)\Sigma^R_{\rm ep}(\omega)]^{-1}G^R(\omega) \tag{S16}$$

and

$$\mathcal{G}^<(\omega) = [I + G^R(\omega)\Sigma^R_{\rm ep}(\omega)]^{-1}G^<(\omega)[I+\Sigma^A_{\rm ep}(\omega)G^A(\omega)]^{-1} - \mathcal{G}^R(\omega)\Sigma^<_{\rm ep}(\omega)\mathcal{G}^A(\omega), \tag{S17}$$

where the product involves matrix multiplication over orbital indices. The impurity and the full Green's functions are then iterated with the self-energies, Eqs. (S14,S15), until convergence is reached.

The current density and the effective temperatures are computed with local Green's functions. The electric current per p_y is computed from the effective chain shown in Fig. S4(b). The current density becomes

$$\begin{aligned}J &= -i\int\frac{dp_y}{2\pi}[-2t\cos(p_ya)]\langle d^\dagger_{\ell A}d_{\ell B} - \text{H.C.}\rangle \\ &= \int\frac{dp_y}{2\pi}\int\frac{d\omega}{2\pi}2t\cos(p_ya)[G^<_{\ell,BA}(\omega) - G^<_{\ell,AB}(\omega)].\end{aligned} \tag{S18}$$

We define the local distribution functions for electron and phonon, respectively, as

$$f(\omega) = -\frac{1}{2}\frac{\text{Im}G^<(\mathbf{r}=0,\omega)}{\text{Im}G^R(\mathbf{r}=0,\omega)}, \quad n(\omega) = \frac{1}{2}\frac{\text{Im}D^<(\omega)}{\text{Im}D^R(\omega)}. \tag{S19}$$

Since these distribution functions reduce to the equilibrium Fermi-Dirac and Bose-Einstein functions at $E=0$, respectively, we extend the nonequilibrium electron and phonon effective temperatures as

$$\begin{aligned}T^2_{\rm el} &= \frac{6}{\pi^2}\int_{-\infty}^{\infty}\omega[f(\omega)-\Theta(-\omega)]d\omega \\ T^2_{\rm ph} &= \frac{6}{\pi^2}\int_0^{\infty}\omega n(\omega)d\omega,\end{aligned} \tag{S20}$$

with the step-function $\Theta(x)$.

REFERENCES

[1] Lee J, Han JE, Xiao S, Song J, Reno JL, Bird JP. Formation of a protected sub-band for conduc-tion in quantum point contacts under extreme biasing. Nature Nanotechnology **9**, 101 (2014).

[2] Ramamoorthy H, Somphonsane R, Radice J, He G, Kwan CP, Bird JP. "Freeing" graphene from its substrate: observing intrinsic velocity saturation with rapid electrical pulsing. Nano Letters **16**, 399 (2016).

[3] Nathawat J, Smithe KKH, English CD, Yin S , Dixit R, Randle M, Arabchigavkani N, Barut B, He K, Pop E, Bird JP. Transient hot-carrier dynamics and intrinsic velocity saturation in monolayer MoS_2. Physical Review Materials **4**, 014002 (2020).

[4] Dixit R, Barut B, Yin S, Nathawat J, Randle M, Arabchigavkani N, He K, Kwan CP, Mishima TD, Santos MB, Ferry DK, Sellers IR, Bird JP. Pulsed studies of intervalley transfer in $Al_{0.35}In_{0.65}As$: A paradigm for valley photovoltaics. Physical Review Materials **4**, 085404 (2020).

[5] Mohammadzadeh A, Baraghani S, Yin S, Kargar F, Bird JP, Balandin AA. Evidence for a thermally driven charge-density-wave transition in 1T-TaS_2 thin-film devices: prospects for GHz switching speed. Applied Physics Letters **118**, 093102 (2021).

[6] Yin S, He K, Randle MD, Barut B, Dixit R, Lipatov A, Sinitskii A, Bird JP. Probing the dynamics of electric double layer formation over wide time scales in the Ionic Liquid DEME-TFSI. Journal of Physical Chemistry C **126**, 1958 (2022).

[7] Li J, Han JE. Nonequilibrium excitations and transport of Dirac electrons in electric-field-driven graphene. Phys. Rev. B **97**, 205412 (2018).

[8] Li, J, Aron, C, Kotliar, G & Han, JE, Electric-Field-Driven Resistive Switching in the Dissipative Hubbard Model. Phys. Rev. Lett. **114**, 226403 (2015).

[9] Aoki, H. et al. Nonequilibrium dynamical mean-field theory and its applications. Rev. Mod. Phys. **86**, 779 (2014).
